# Analysis Model for Infant Incubator Adverse Events Using Retrieval-Augmented Generation Combined With Dual-Adapter Fine-Tuning: Development and Evaluation Study

**DOI:** 10.2196/83745

**Published:** 2026-03-31

**Authors:** Wenke Xia, Wanting Zhu, Tianchun Li, Li Wang, Weiqi Li, Peiming Zhang

**Affiliations:** 1 School of Health Science and Engineering University of Shanghai for Science and Technology Shanghai China; 2 Center for Drug Reevaluation of Henan Zhengzhou China

**Keywords:** adverse events in medical devices, infant incubators, large language models, fine-tuning techniques, retrieval-augmented generation

## Abstract

**Background:**

Infant incubator adverse events refer to various harmful incidents that occur during the normal use of marketed infant incubators and result in, or may result in, bodily harm. In recent years, however, the number of reported adverse events has continued to rise. This trend has made the monitoring of infant incubator adverse events time-consuming and labor-intensive when relying solely on manual processing by medical device adverse-event monitoring personnel. Meanwhile, general-purpose large language models (LLMs) still face domain knowledge gaps and hallucination issues in specialized fields. Through fine-tuning, LLMs can be adapted to specific application scenarios, while retrieval-augmented generation (RAG) enhances their ability to handle knowledge-intensive tasks. Therefore, LLMs that integrate these 2 technologies hold significant potential for addressing monitoring challenges.

**Objective:**

This study aims to enhance the intelligent monitoring of adverse events related to medical devices by integrating RAG with dual-adapter fine-tuning to construct an adverse-event analysis model for infant incubators. The model enables an integrated workflow centered on extraction and analysis, encompassing structured extraction of adverse events, narrative analysis, and regulatory question answering.

**Methods:**

This study leveraged adverse-event data from Chinese infant incubators to construct a high-quality dataset through prompt engineering. Technologically, it combined 2 parameter-efficient fine-tuning methods—low-rank adaptation and infused adapter by inhibiting and amplifying inner activations—to achieve efficient adaptation on the Qwen2-7B base model. Simultaneously, it introduced the FINBGE embedding model with supervised contrastive semantic optimization to build a knowledge retrieval system that mitigates hallucination.

**Results:**

This study comprises 1565 pediatric disease question-and-answer entries from PediaBench, 2530 specific corpora on adverse events in infant incubators, and 1488 regulatory corpora. Extensive experiments demonstrate the superiority of this analytical model across various metrics. Under the same experimental conditions, the element recall rate reaches 0.815, the accuracy of infant incubator adverse event analysis is 0.898, and the accuracy in regulatory clause question-answering tasks attains 0.938.

**Conclusions:**

The analytical model proposed in this study demonstrates significant advantages in analyzing adverse events related to infant incubators, while also achieving substantial improvements in text generation metrics. When combined with RAG, it not only effectively mitigates hallucination but also enhances knowledge timeliness. This study employs the Qwen model as its foundational framework, leveraging large-model fine-tuning and RAG to achieve intelligent analysis of high-risk medical device adverse-event monitoring data. It demonstrates the feasibility of implementing intelligent medical device regulation within the Qwen ecosystem.

## Introduction

### Background

Collecting and analyzing adverse event monitoring data for high-risk medical devices is a crucial means of ensuring their safety and effectiveness [1]. Currently, medical device regulatory authorities have widely adopted electronic monitoring systems, which not only enhance data collection efficiency and convenience but also lead to a sustained increase in the volume of adverse event reports submitted [2]. Badnjević et al [3] found that despite clear regulatory mandates for postmarket surveillance obligations across countries, issues persist in adverse event reporting, including inconsistent standards, noncomparable data, and fragmented oversight. In China, infant incubators are classified as class III high-risk medical devices. Adverse event monitoring for these products involves interdisciplinary knowledge integration across multiple fields. Challenges include large volumes of adverse event data, predominantly in unstructured and nonstandardized formats [4], as well as significant variation in data quality reported by health care institutions [5]. These factors substantially increase the workload and complexity of analyzing and processing reports. Recent innovations in natural language processing technology offer new possibilities for balancing efficiency and professionalism. Ruseva et al [6] proposed a deep learning model based on the TensorFlow framework, demonstrating strong performance in assessing adverse drug reaction risks and enhancing postmarketing drug surveillance capabilities. Hauben [7] developed artificial intelligence (AI)–based methods to enhance drug-drug interaction detection and prediction. Borjali et al [8] integrated deep learning to accurately and promptly detect adverse medical events from free-text medical narratives.

While traditional deep learning models can perform text classification, they lack long-range contextual associations [9] and causal reasoning capabilities [10], exhibit weak generalization, and demonstrate poor transferability between events [11]. Lu et al [12] found that Transformer models consistently outperformed traditional convolutional neural network models in nearly all medical text classification scenarios, with convolutional neural networks demonstrating acceptable performance only when categories were balanced. Naik et al [13] highlighted that adapting event extractors to new medical domains suffers from covariate shift issues, rendering direct transfer ineffective and necessitating specialized domain adaptation techniques.

Although large language models (LLMs) can provide solutions to core challenges in medical device adverse event monitoring, pretraining foundational models from scratch for vertical domains incurs prohibitively high costs. For instance, first, training a model with tens of billions of parameters requires multiple graphics processing units and months of computational time [14], demanding substantial computational resources and time investment. Second, pretraining often requires datasets in the gigabyte or even terabyte range [15], while vertical domain data are typically strictly confidential [16]. Publicly available data may constitute less than 0.1% of pretraining requirements, and the processes of data collection, cleaning, annotation, and storage involve substantial human and material resources. Finally, pretraining imposes steep technical barriers, demanding both deep algorithmic engineering expertise and the development of large-scale distributed training frameworks [17]—a burden beyond the reach of most small-to-medium teams.

Therefore, this study constructs an infant incubator adverse event analysis model by fine-tuning LLMs. Methods such as low-rank adaptation (LoRA) and infused adapter by inhibiting and amplifying inner activations, that is, (IA)^3^, require only minor weight updates, preserving general language prior knowledge while incorporating domain-specific expertise [18]. However, for tasks involving the analysis of adverse events related to infant incubators—which are complex, densely populated with specialized terminology, and characterized by imbalanced samples—a single method has inherent limitations. LoRA excels at capturing long-range contextual dependencies and temporal event correlations through low-rank updates, but its global low-rank matrix may lack sufficient feature resolution when handling rare fault descriptions or subtle terminological variations. Meanwhile, (IA)^3^ enables fine-grained control over the strength of specific features through dynamic scaling of activation values, but it struggles to model long-range semantic relationships across sentences or paragraphs. Neither approach alone can comprehensively address these challenges.

To this end, this study proposes a “dual-adapter joint fine-tuning” architecture. Its novelty lies not in simple superposition but in a hierarchical division of labor and collaboration: the LoRA component focuses on modeling logical chains and long-range dependencies within adverse events, while the (IA)^3^ component performs local feature enhancement and normalization on terminology and key entities to suppress noise. Coupled through a dynamic weight transfer mechanism, this approach enables the model to achieve deep adaptation to vertical domain knowledge while preserving general medical semantic understanding. Although vertical domains often lack extensive data, Wang et al [19] demonstrated the feasibility of studying small-sample adverse event data in medical devices when investigating knowledge transfer and calibration for small-sample medical signals (epileptic electroencephalography).

High-quality and task-relevant data form the cornerstone for ensuring that fine-tuned models achieve the expected performance, accuracy, and specific behavioral patterns. Data quality directly determines the nuances learned by the model, the biases it may inherit or mitigate, and its generalization capabilities [20]. The data source used in this study comprises nonpublic adverse event data from infant incubators within China’s Medical Device Adverse Event Monitoring System (the details of adverse events are shown in Multimedia Appendix 1). This dataset authentically captures adverse events generated by infant incubator use in medical institutions in recent years, encompassing complex and diverse semantic expressions within the domain. Real-world noise reflects the challenges of practical application scenarios; mild noise can actually compel the model to learn deeper semantic features rather than relying on spurious patterns in the data, thereby enhancing model robustness [21].

It is important to note that even after model fine-tuning, hallucination persists. From a computational theory perspective, all AI models—including current mainstream LLMs—are fundamentally probabilistic mathematical function approximators [22]. These models map inputs to output spaces through complex parameter matrix operations, generating outputs that are essentially responses sampled from high-dimensional probability distributions. Retrieval-augmented generation (RAG) significantly mitigates this issue. It is an AI technique that combines information retrieval technology with language generation models [23]. This approach enhances the model’s capability to handle knowledge-intensive tasks by retrieving relevant information from external knowledge bases and feeding it as prompts to LLMs.

### Related Work

In recent years, the rapid advancement of AI technology has increasingly highlighted the application value of large-scale pretrained models across multiple professional domains. Advanced models such as GPT and Bidirectional Encoder Representations from Transformers (BERT), with their exceptional language comprehension and generation capabilities, have demonstrated significant application potential in the health care sector. Research data indicate that models based on the Transformer architecture possess unique advantages in medical text processing: Kim et al [24] confirmed that domain-adapted BERT variants perform well in predicting appropriate medical departments based on patient symptom descriptions and in answering specialized medical questions. Zhang et al [25] utilized LLMs for medical record generation tasks, saving physicians approximately 0.5-1 hour of work time daily.

Notably, the application scope of such models has expanded beyond text processing into medical devices and regulatory domains. Tang et al [26] highlighted that the global AI medical device industry is experiencing robust growth, while the urgent need for regulatory oversight is becoming increasingly apparent—a trend expected to intensify. For instance, Kim et al [27] developed a deep learning predictive model to rapidly and accurately detect mechanical ventilation requirements in neonatal intensive care units using electronic health records; Matheny et al [28] systematically explored how applying LLMs to the US Food and Drug Administration’s (FDA) medical product safety surveillance system enhances the identification and assessment of adverse drug events.

Regarding fine-tuning medical LLMs, multiple studies have explored application methods. For instance, Jia et al [29] employed LoRA for instruction fine-tuning of pretrained LLMs, ultimately enhancing the automated generation of children’s health science resource labels. Abbasian et al [30] utilized electronic health records from patients with diabetes for P-tuning and LoRA fine-tuning of large models, opening new avenues for diabetes management and improving therapeutic efficacy. Integrating large models with RAG has also become a research hotspot. Hu et al [31] introduced a pioneering personalized dementia care approach by integrating GPT-4, fine-tuned and RAG-enabled, with a vector database. Kulshreshtha et al [32] enhanced a medical chatbot based on the Llama source model using RAG methods to generate reliable, contextually relevant responses, thereby improving clinical judgment and reducing health care workers’ workload.

## Methods

### Overview

**Figure 1:**
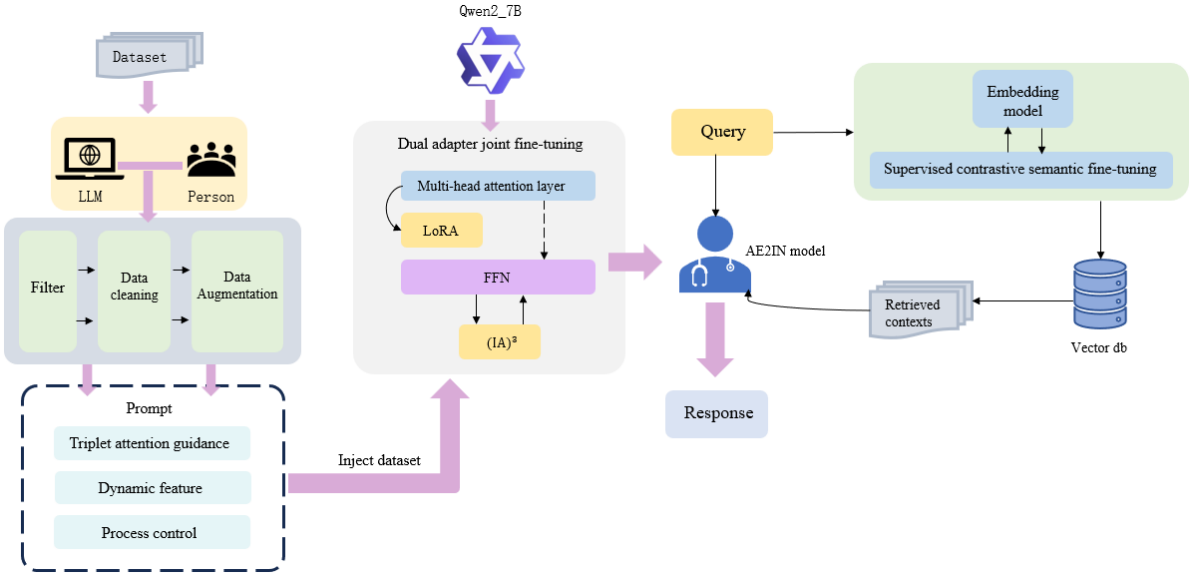
Framework diagram of the AE2IN adverse event analysis model for infant incubators. The preprocessing layer cleans the data, normalizes terminology, and enhances adverse event reports. The knowledge enhancement layer enables collaborative analysis through external knowledge injection and large-model semantic reasoning, using a Qwen model fine-tuned with dual adapters alongside a fine-tuned embedding model. FFN: feedforward network; (IA)3: Infused Adapter by Inhibiting and Amplifying Inner Activations; LLM: large language model; LoRA: low-rank adaptation.

The adverse event analysis model AE2IN proposed in this study for infant incubators comprises 2 core modules: a data preprocessing layer and a knowledge-enhanced analysis layer. The specific framework is illustrated in Figure 1.

At the data preprocessing layer, the system first standardizes input adverse event reports for infant incubators through data filtering, cleaning, and augmentation. The data standardization, cleaning, and augmentation processes for this study’s training dataset leveraged GPT-4 as a teacher model for auxiliary processing. Through knowledge distillation in a teacher-student paradigm, it provided high-quality training samples for subsequent domain-specific fine-tuning of the Qwen2_7B model. Standardization during conventional inference fully utilizes specialized medical dictionaries and regular expression rules to ensure accurate recognition of diverse terminology. The preprocessed structured data are subsequently fed into the knowledge-enhanced analysis layer.

The knowledge-enhanced analysis layer serves as the system’s core, employing a dual-path parallel processing mechanism. On the one hand, supervised comparison-based semantic optimization fine-tunes the embedded model FINBGE to vectorize queries and retrieve the most relevant clause content from the medical device regulatory knowledge base. On the other hand, the Qwen2_7B model, jointly fine-tuned via dual adapters, performs deep semantic understanding of the input text. Notably, this model innovatively combines 2 parameter-efficient fine-tuning techniques: LoRA and (IA)^3^.

### Fine-Tuning

#### Dual-Adapter Fine-Tuning

**Figure 2:**
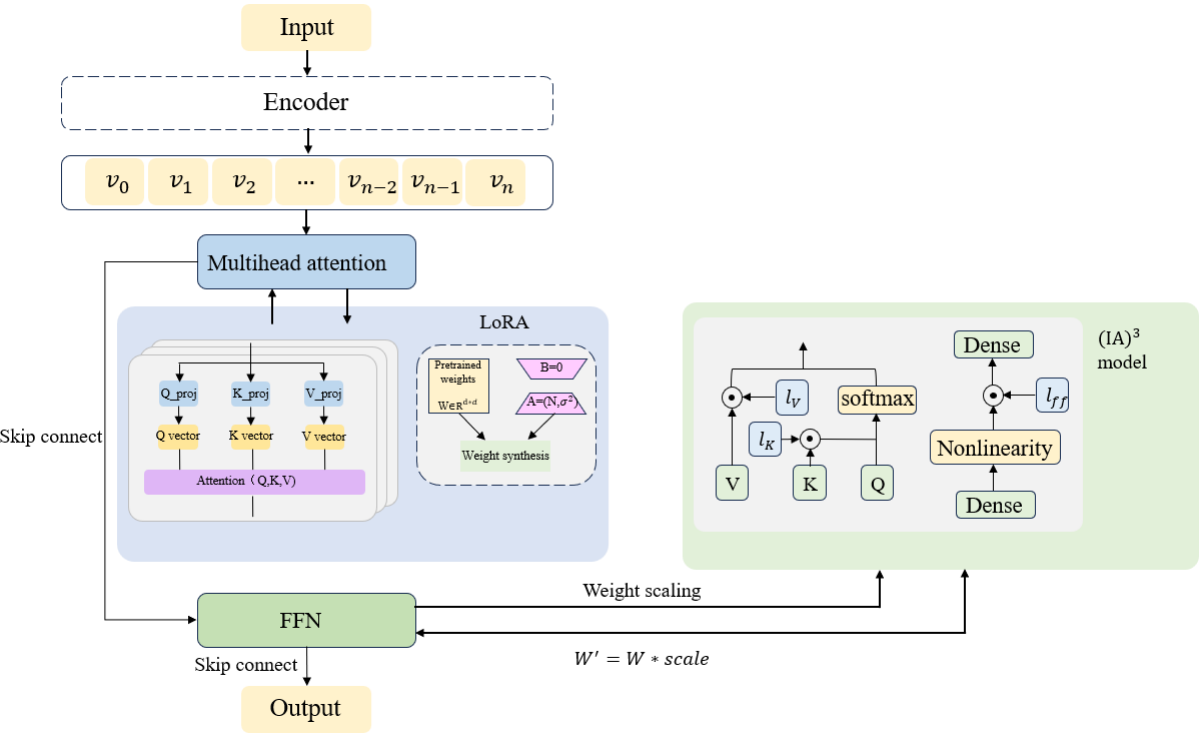
Dual-adapter fine-tuning framework diagram. The architecture is built on the Qwen2_7B foundation model and integrates 2 parameter-efficient methods: low-rank adaptation (LoRA) and Infused Adapter by Inhibiting and Amplifying Inner Activations (IA)3. The diagram illustrates the core design, with LoRA applied to the query/value projection matrices in the Transformer attention mechanism and (IA)3 applied to the feedforward network (FFN). It also depicts the fine-tuning strategy, where pretrained weights are frozen and only adapter parameters are updated, highlighting key processes such as attention computation, weight composition, and feature scaling.

This study designed a dual-adapter joint fine-tuning method that combines LoRA and (IA)^3^, 2 parameter-efficient fine-tuning techniques. LoRA focuses on optimizing the Q (query)/V (value) projection matrices within the attention mechanism through low-rank matrix updates (*r*=32), effectively capturing contextual dependencies in descriptions of adverse events in infant incubators. Meanwhile, (IA)^3^ refines the feature representations of the feedforward network by learning feature scaling coefficients without introducing additional parameters, thereby enhancing the representation of abnormal features and the generation of handling suggestions. Mathematically, this can be expressed as a composite transformation:

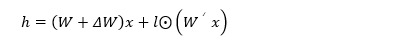



where *W* represents the original pretrained weights, Δ*W* is the low-rank update matrix introduced by LoRA, composed of the product of 2 small matrices A and B, and *l* is the feature scaling vector learned by (IA)^3^. This architecture retains LoRA’s advantage in pattern discovery—learning domain-specific attention patterns through low-rank space—while integrating the precise feature selection control of (IA)^3^. The overall framework is illustrated in Figure 2.

The domain-adaptive training strategy employs a dynamic weighting mechanism to balance domain-specific data with general-purpose corpora. Its objective function is defined as follows:

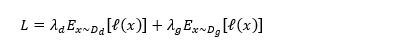

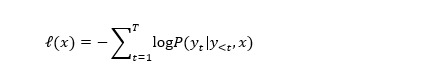





where *λ_d_* is the domain data weight coefficient; *λ_g_* is the general data weight coefficient, with a ratio of 3:1; *D_d_* and *D_g_* represent the domain dataset and general dataset, respectively; 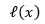 denotes the loss function for a single sample; *y_t_* is the *t*th token in the target sequence; *y*_<_*_t_* indicates the historical output sequence; and *T* is the target sequence length.

By employing a 3:1 weighted sampling mechanism, domain data receive high-frequency exposure to accelerate the model’s mastery of infant incubator adverse event norms, while continuous infusion of general data maintains foundational medical semantic understanding.

This method can be divided into 2 stages. The first stage focuses on optimizing the attention mechanism. By utilizing low-rank matrix updates, it establishes cross-layer semantic associations for factors in the Q/V projection matrix that contribute to adverse events in infant incubators. This step notably enhances the model’s ability to understand temporal patterns in infant incubator adverse events. It can be conceptualized as the model’s capability to accurately capture causal chains such as “temperature alarm → skin temperature probe failure → component inspection.” The second stage specifically adjusts the feature representations in the feedforward network. By learning scaling coefficient vectors, it finely controls the generation intensity of infant incubator adverse event terminology and the standardization of subsequent handling recommendations. For example, operations such as “Immediately replace the incubator, monitor the infant’s temperature and pulse oxygen saturation, and report to the equipment department for repair” fall under the follow-up actions category, while information such as “Equipment model YP-90B, root cause analysis of the fault” is accurately categorized under the additional information category. This ensures that the model’s output maintains both professional precision and compliance with medical documentation standards.

Knowledge inheritance between stages is achieved through dynamic weight transfer—using attention patterns obtained from LoRA training as initialization priors for the (IA)^3^ stage. This strategy converges faster than parallel training while preserving model generalization capabilities and notably enhancing domain-specific task performance. It effectively overcomes the limitations of either approach: fine-grained feature variations that are difficult for LoRA to capture are compensated by (IA)^3^, while long-range dependency modeling—a weakness of (IA)^3^—is supplemented by LoRA.

#### Supervised Contrastive Semantic Optimization Fine-Tuning

**Figure 3:**
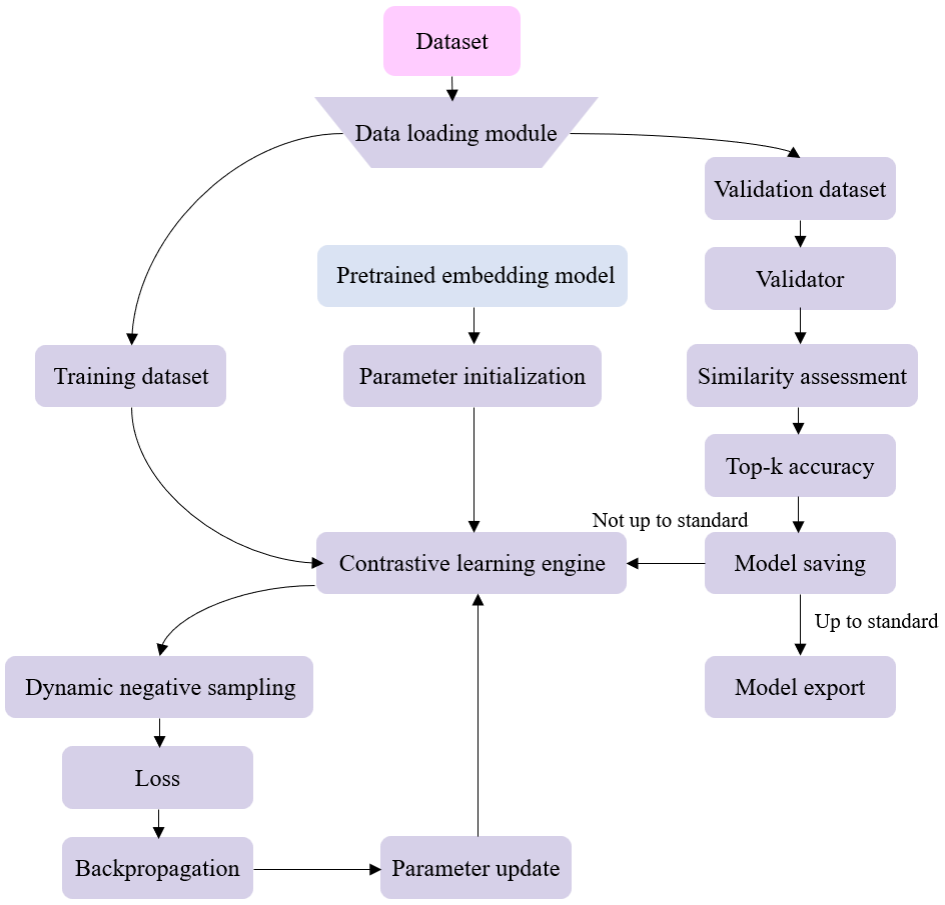
Framework diagram of the supervised contrastive semantic optimization fine-tuning process. The model performs comparative learning and parameter updates using both the training and validation datasets.

In specialized domains, general-purpose embedding models often struggle to accurately capture subtle distinctions between professional terminology, leading to similar yet unrelated concepts being overly close in vector space [33]. Therefore, this paper proposes a supervised contrastive semantic optimization method to fine-tune embedding models. The fine-tuning workflow framework is illustrated in Figure 3.

By constructing an end-to-end fine-tuning pipeline, we achieve efficient domain adaptation for pretrained embedding models. Utilizing the Sentence Transformers Finetune Engine framework, we combine contrastive learning mechanisms with domain knowledge injection. This enables the model to accurately capture domain-specific semantic association patterns while preserving its original semantic understanding capabilities. The core training objective is mathematically expressed as follows:

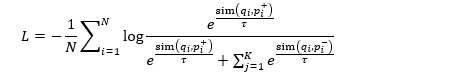



where *q_i_* is the embedding vector for query *i*; *p_i_*^+^ and *p_i_*^–^ are the positive and negative samples associated with *q_i_*, respectively; *τ* is the temperature coefficient controlling the sharpness of the distribution; *N* denotes the number of queries in the batch; and *K* represents the number of negative samples corresponding to each query. Cosine similarity establishes the relevance metric between queries and documents. The temperature parameter *τ* controls the sensitivity of similarity scores. The numerator term *e*[sim(*q_i_*, *p_i_*^+^)/*τ*] generates centripetal force, drawing relevant documents closer to the query point in vector space. The denominator term 
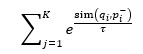
 generates a repulsive field, pushing irrelevant documents away from the query point’s semantic neighborhood.

Beyond reconstructing the semantic space, feature space calibration requires an embedding projection transformation, formulated as:

*h* = LayerNorm[*W*_2_·GELU(W_1_*x* + *b*_1_) + *b*_2_]

*W*_1_ and *W*_2_ form a parameterized transformation matrix that projects the original embedding onto a subspace more suitable for the current task. The GELU function introduces a smooth threshold, preserving important features while filtering out noise. LayerNorm maintains numerical stability, ensuring comparability between embeddings from different domains.

### Infant Incubator Adverse Event Evaluation Indicators

When analyzing adverse events involving infant incubators, core elements are of paramount importance. Yan and Huifang [34] proposed analyzing both the manifestations and root causes within adverse event data for infant incubators. Kaur et al [35] suggested describing the implemented control measures. Additionally, according to the writing requirements of the guidelines issued by the National Medical Products Administration, adverse event reports typically require information such as location, time, source, injury manifestation, device malfunction, cause analysis, and specific control measures. Accordingly, we summarize the core and secondary elements of infant incubator adverse events. The core elements are the “occurrence date” of the adverse event, the “abnormal condition” of the infant incubator, and the “follow-up measures” for risk control. Secondary elements include the “cause of failure” and the “risk triggered” by the infant incubator. Beyond these core and secondary elements, any additional information captured from the original text is categorized as supplementary notes.

Therefore, during testing experiments, in addition to employing general evaluation metrics, we have developed specialized evaluation indicators for adverse events in infant incubators: the element recall rate and the information density index of adverse events with correction factor (IDIAE). The formulas are as follows:

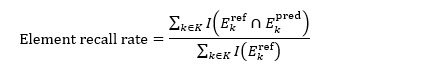



where *K* denotes the core feature set; *E_k_*^ref^ represents feature *k* in the reference text; *E_k_*^pred^ denotes feature *k* in the predicted text; and *I* is the indicator function. During computation, text segments *E_k_*^ref^ and *E_k_*^pred^ corresponding to the 3 core elements are extracted from the reference text and prediction text, respectively. Subsequently, a hierarchical matching assessment is performed: an exact match is determined by checking whether the normalized reference text is fully contained within the prediction text. If the exact match fails, the similarity ratio based on edit distance between the 2 texts is calculated. If this ratio exceeds the threshold of 0.8, the segment is deemed a match.

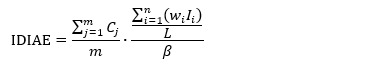



*β =* 1 + *α*·(*N*_red_/10) + γ·*M*_error_

where *C_j_* represents the coverage status of the *j*th standard report field; *m* denotes the total number of required fields; *w_i_* is the weight coefficient for the *i*th category of information (core element weight is 1.0, secondary requirement weight is 0.8); *β* is the text redundancy coefficient, and the redundancy coefficient incorporates a redundancy word penalty weight *α* to assess whether the model’s response is excessively verbose; *N*_red_ indicates the number of irrelevant characters; *γ* is the terminology error penalty weight used to evaluate whether the model captures professional terminology correctly; and *M*_error_ represents the number of errors.

**Figure 4:**
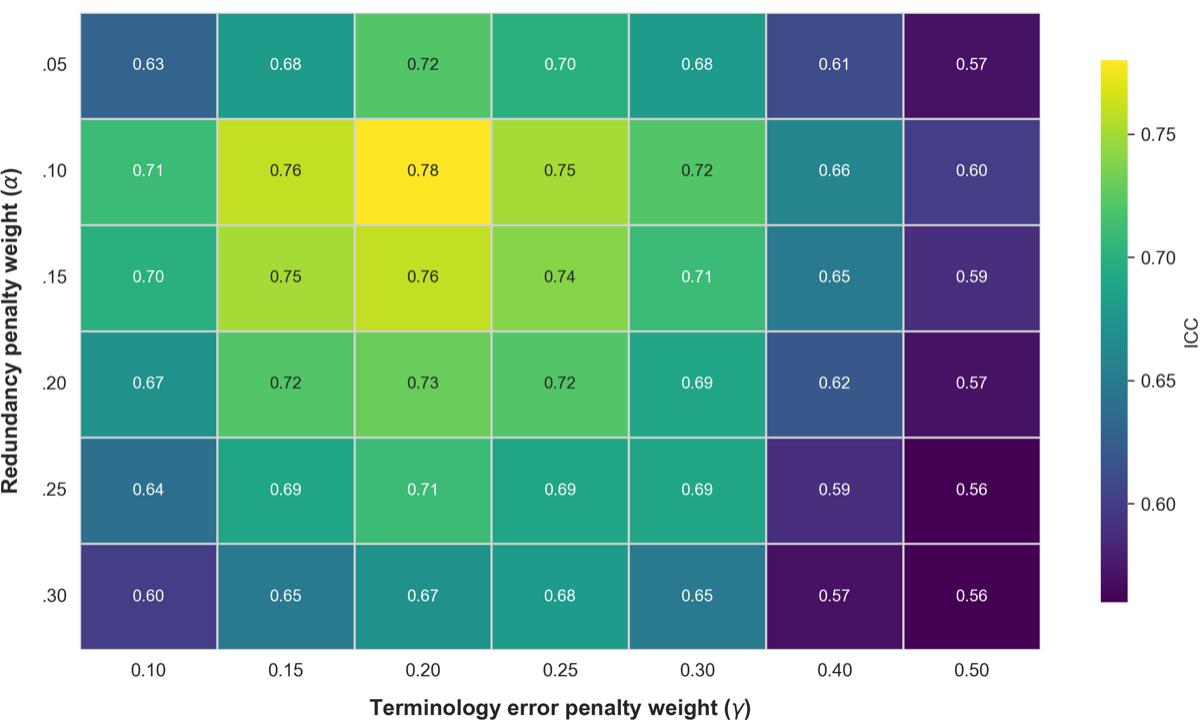
Sensitivity heatmap of Information Density Index of Adverse Events (IDIAE) metric weights (intraclass correlation coefficient [ICC] values). The experiment used 80 randomly selected adverse event reports from infant incubators, with expert blinded scoring as the gold standard. The x- and y-axes represent the redundancy penalty weight (α) and the terminology error penalty weight (γ), respectively. Color blocks indicate the ICC values for each weight combination, enabling identification of the globally optimal parameter setting.

**Figure 5:**
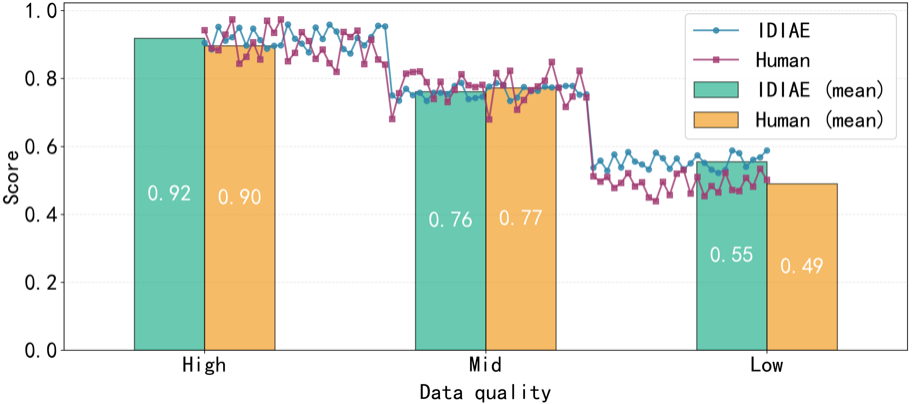
Comparison of Information Density Index of Adverse Events (IDIAE) and manual scoring integrity. The figure compares completeness scores and their mean values between the IDIAE metric and human expert ratings across 3 data groups with varying quality levels.

**Figure 6:**
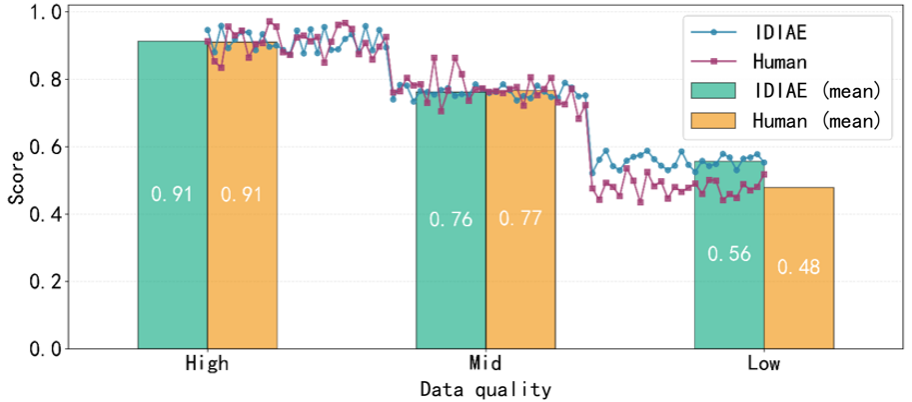
Accuracy comparison between Information Density Index of Adverse Events (IDIAE) and human scoring. The figure compares accuracy scores and their mean values between the IDIAE metric and human expert ratings across 3 data groups with varying quality levels.

To determine the globally optimal combination of *α* and *γ* and to validate the reliability of the IDIAE metric, this study randomly selected 80 adverse event reports for infant incubators. These were stratified into high-, medium-, and low-quality grades, with 26, 28, and 26 reports, respectively. Experts were invited to rescore the reports under blinded conditions. As shown in Figure 4, varying only the *α* and *γ* values revealed that the intraclass correlation coefficient peaked at 0.78 (95% CI 0.72-0.83) when *α*=.1 and *γ*=0.2. This indicates a strong positive correlation between IDIAE scores and expert ratings under this weighting configuration (correlation coefficient *r*=0.82, *P*<.001), representing the globally optimal combination. To further validate IDIAE reliability, after fixing the weights (*α*=.1, *γ*=0.2), analyses across high-, medium-, and low-quality grades showed that the mean IDIAE scores for all 3 groups in Figures 5 and 6 consistently aligned with expert-rated mean trends for both completeness and accuracy. The classification consistency *κ* coefficient reached 0.71. These results demonstrate that the IDIAE metric effectively reflects expert criteria for assessing report completeness, exhibiting good reliability and clinical applicability.

### Prompt Engineering

**Figure 7:**
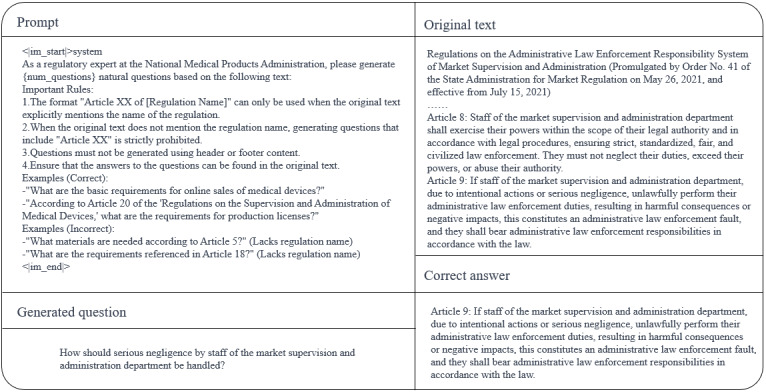
Prompt for generating fine-tuning datasets for embedding models.

**Figure 8:**
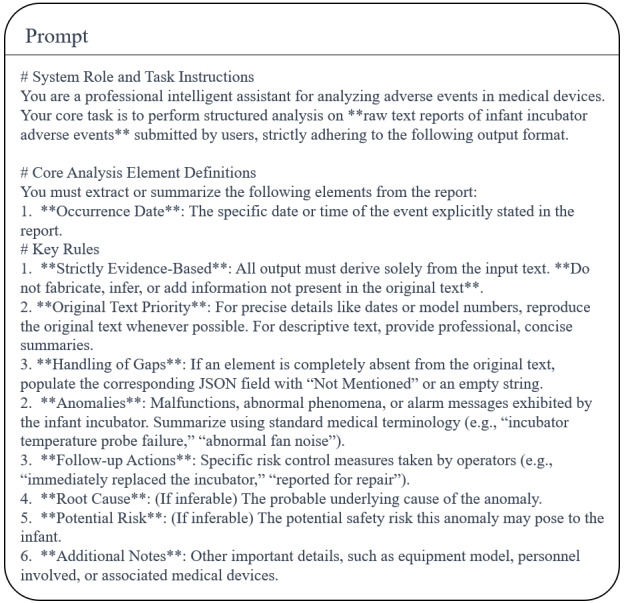
System prompt for the inference phase. The template defines the model’s role, output specifications, and core constraints for adverse event analysis and regulatory questions and answers in infant incubators. It governs output format, professional compliance, and content boundaries to ensure responses meet specialized regulatory requirements for medical device adverse events.

**Figure 9:**
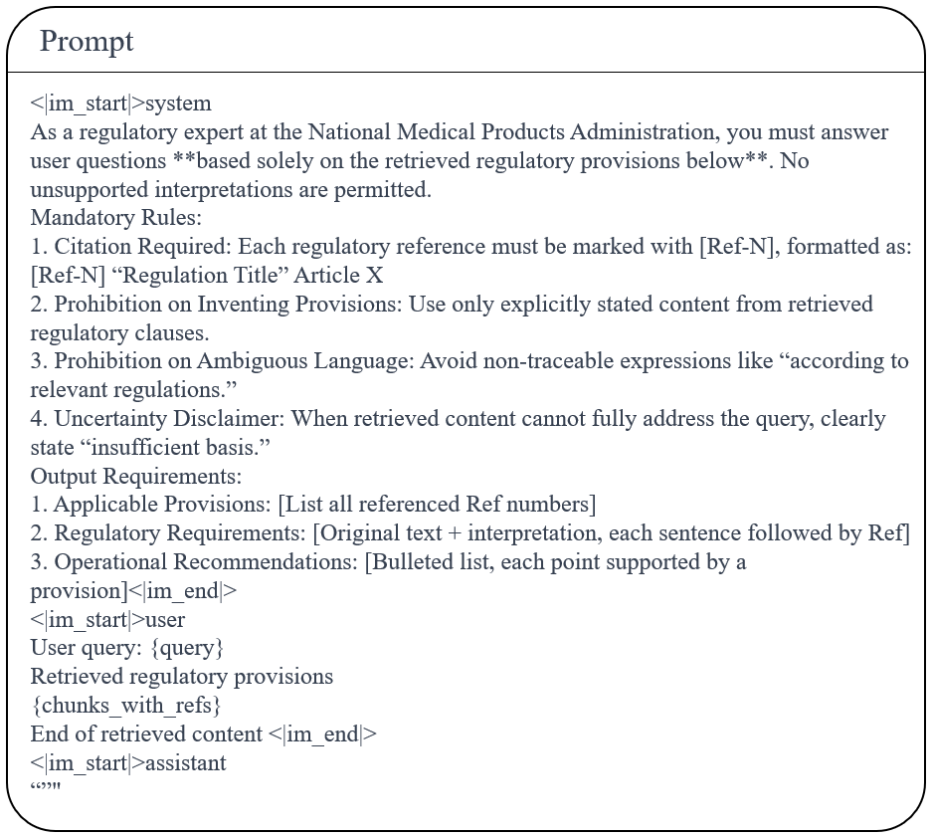
Retrieval-augmented generation retrieval prompt template. Designed for a regulatory expert role aligned with the National Medical Products Administration, it defines 4 mandatory rules and standardized output requirements: conclusions must be based solely on retrieved regulatory provisions; each interpretation must include source citations; fabrication of provisions is prohibited; and any uncertainty must be labeled as “insufficient basis.” The template takes user queries and retrieved regulatory texts as input and produces standardized regulatory question-and-answer outputs.

To efficiently align the model with the desired training outcomes, this study designs 3 prompts for different scenarios, as shown in Figures 7-9. Figure 7 is used to assist in generating a suitable fine-tuning dataset. Figure 8 presents the system prompt embedded in the inference stage, aiming to standardize the model output. The retrieval prompt integration employs a structured constraint template, as shown in Figure 9. System instructions enforce that the model must base its responses solely on retrieved content, annotating each sentence with a source identifier [Ref-N] after interpretation. Each retrieved chunk is organized according to a fixed format. The prompt template constrains model behavior through 3 core rules:

- It explicitly prohibits vague references such as “Article X,” mandating explicit linkage to specific regulatory document names and clause numbers to prevent cross-document clause confusion.
- It requires declarations of “insufficient basis” for uncertain content, thereby eliminating model speculation or the fabrication of nonretrieved clauses.
- Regulatory requirement sections must alternate between verbatim citations and interpretations, with each interpretive sentence immediately followed by a [Ref-N] annotation.

### Optimal Parameter Configuration

**Figure 10:**
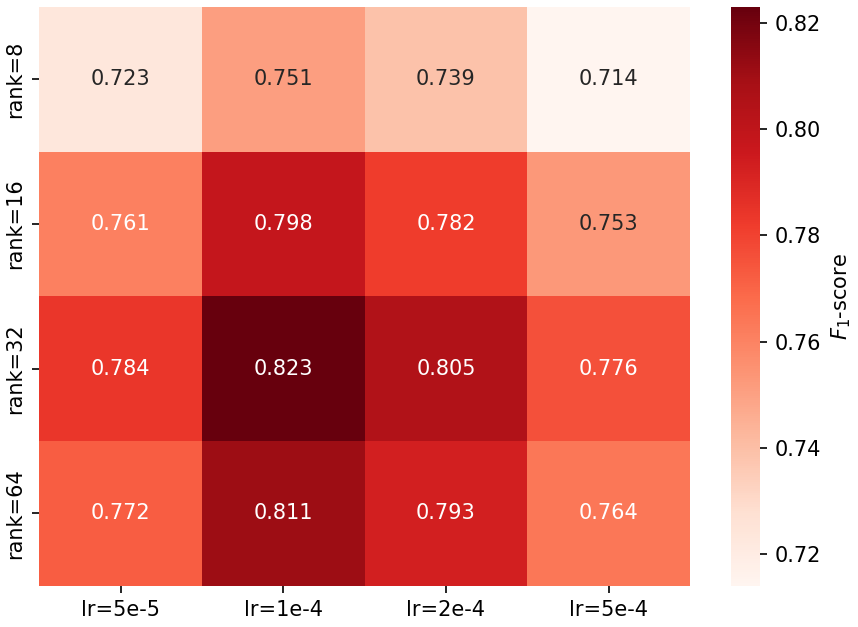
Hyperparameter sensitivity heatmap. The figure shows variation in F1-scores on the test set for the infant incubator adverse event question-answering task as a function of low-rank adaptation rank and learning rate, evaluated via a 4 × 4 grid search.

In typical small-sample medical data analysis tasks, *r*=32 has been validated as an effective LoRA range [36]. Before experimentation, we initially favored selecting *r*=32. However, to rigorously validate the impact of rank-learning rate combinations on the performance of the infant incubator adverse event question-answering task, we conducted a 4 × 4 grid scan while fixing all other hyperparameters. The resulting *F*_1_-scores are visualized as a heatmap, as shown in Figure 10. The heatmap shows that the *F*_1_-score peaks at 0.823 at rank=32 and lr=1 × 10^–4^, then decreases toward the surrounding edges, exhibiting an overall bell-shaped “medium-high-medium” distribution. This phenomenon occurs because when the rank is too low, the low-rank matrix lacks expressive power and cannot fully capture the long-range dependencies and medical terminology details in event descriptions; when the rank is too high, the number of parameters increases, which can lead to overfitting on domain data and weaken generalization. Regarding the learning rate, 5 × 10^–5^ converges too slowly, whereas 5 × 10^–4^ has an excessively large step size, making it difficult for the model to stabilize at the optimal weights. By contrast, 1 × 10^–4^ strikes a balance between convergence speed and stability. Therefore, the experiment ultimately adopted the combination of rank=32 and lr=1 × 10^–4^.

**Figure 11:**
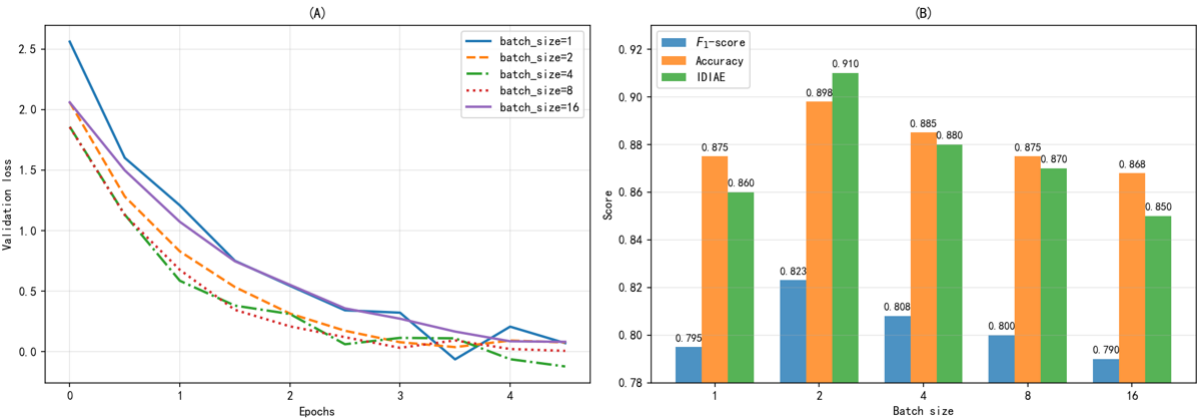
Batch size impact analysis. The figure examines how different training batch sizes affect the AE2IN model’s training stability and final performance. (A) Training loss curves across epochs for various batch sizes, with epochs on the x-axis and loss on the y-axis. (B) The final F1-score, accuracy, and Information Density Index of Adverse Events (IDIAE) metric for each batch size, with batch size on the x-axis and metric values on the y-axis.

To investigate the impact of batch size on training stability and performance, we conducted experiments while fixing the other hyperparameters. As shown in Figure 11A, when batch size is 1, the loss curve oscillates notably and converges slowly due to high gradient update noise; a batch size of 2 (the configuration selected for this study) achieves an optimal balance between training stability and convergence speed, with a smoothly declining loss curve and the lowest final convergence value; increasing the batch size to 4 or 8 yields a more stable training process but slightly slower convergence; a batch size of 16 fails to iterate sufficiently due to graphics processing unit memory constraints. Figure 11B further quantifies the final performance metrics across different batch sizes: a batch size of 2 achieves peak values for *F*_1_-score, accuracy, and IDIAE, showing average improvements of 4.0% and 3.4%, compared with batch sizes of 1 and 8, respectively. Therefore, the final experimental configuration adopts a batch size of 2.

**Figure 12:**
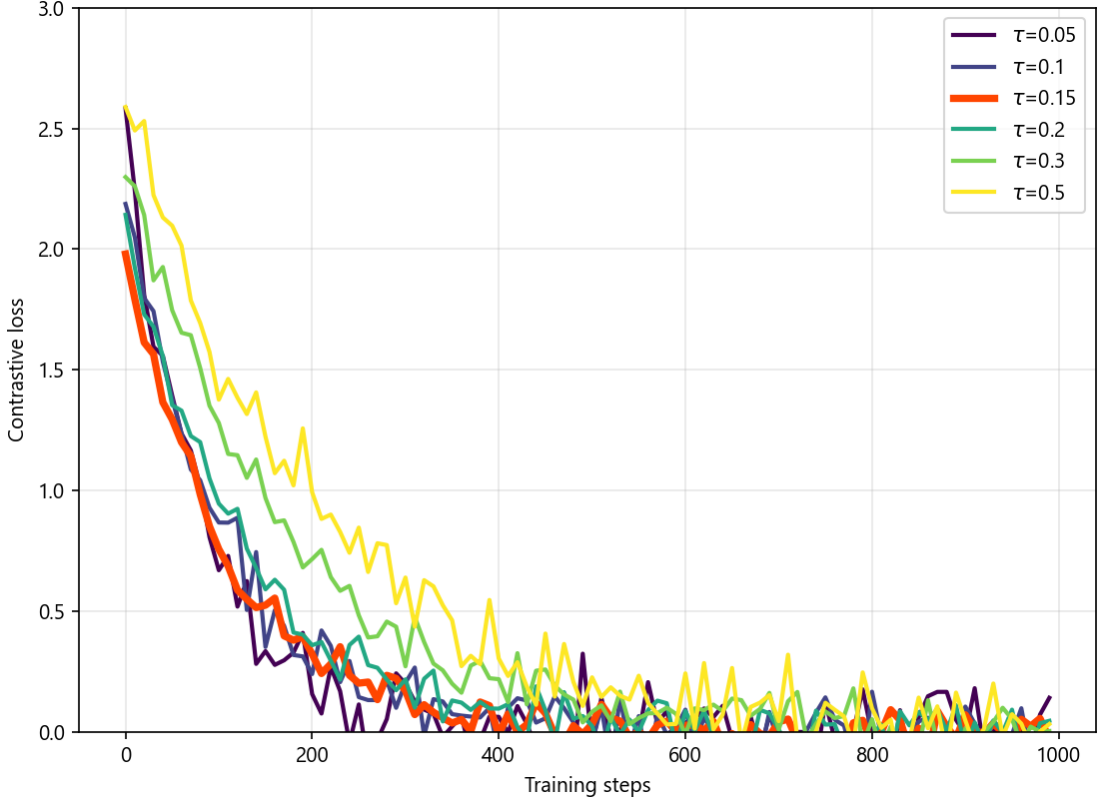
Effect of the temperature coefficient (τ) on supervised contrastive learning. The figure shows how different τ values influence convergence of the training loss during supervised contrastive semantic optimization fine-tuning of the embedding model. The x-axis represents training steps, and the y-axis shows the contrastive learning loss.

In supervised contrastive semantic optimization, the temperature coefficient *τ* controls the sharpness of the similarity distribution. To achieve optimal results, we fixed the other parameters and conducted experiments on the temperature coefficient. As shown in Figure 12, when *τ*=0.05, the similarity distribution becomes excessively sharp, causing the loss curve to oscillate and converge slowly. At *τ*=0.15 (the value selected for this study), the loss curve declines smoothly and rapidly to a stable region. When *τ* increases beyond 0.3, the similarity distribution becomes overly smooth, notably slowing loss convergence. Therefore, *τ*=0.15 is determined to be the optimal configuration for medical device regulatory retrieval tasks.

### Ethical Considerations

Our data originate from China’s medical device regulatory authorities. The data consist of medical device adverse event reports (similar to the US FDA’s Manufacturer and User Facility Device Experience [MAUDE] database). The content primarily includes failure information recorded during device use. These data contain no patient information; therefore, formal ethics approval is not required. This project was jointly completed with regulatory agencies under government-funded research initiatives. All data were authorized, and the research complies with relevant regulations.

## Results

### Experimental Data

This large-model experimental dataset comprises general-domain data and domain-specific data. The general-domain data originate from the PediaBench dataset publicly released by the State Key Laboratory of Public Big Data at the School of Computer Science and Technology, Guizhou University, China. It contains 1565 question-and-answer entries covering common pediatric diseases such as neonatal disorders, pediatric infectious diseases, pediatric respiratory diseases, and pediatric primary care and developmental behavioral disorders. Domain-specific data originate from private adverse event data on infant incubators within China’s Key Monitoring Information System for Medical Device Adverse Events. After manual and GPT-4 screening, data cleaning, and augmentation, combined with prompt engineering, the data were processed into a specialized question-and-answer dataset on infant incubator adverse events, totaling 2530 entries (Table 1).

**Table 1:** Fine-tuning the Qwen model dataset sources and scale.

Dataset	Data source	Data volume
PediaBench	Public dataset from the State Key Laboratory of Public Big Data, School of Computer Science and Technology, Guizhou University, China	1565
Infant Incubator Adverse Event Questions and Answers	Adverse event data on private infant incubators in China’s Key Monitoring Information System for Medical Device Adverse Events	2530

We split the data at the adverse-event report level: all question-and-answer pairs derived from the same report were assigned entirely to either the training, validation, or test set, ensuring that no report appears in more than one set. This experimental dataset allocates 60% (2457/4095) to the training set, 30% (1229/4095) to the test set, and 10% (409/4095) to the validation set.

The knowledge base data originate from regulatory documents issued by the China National Medical Products Administration. We selected administrative regulations and departmental rules to form the regulatory provisions knowledge base, comprising a total of 29 documents. The fine-tuning dataset for the embedding model is derived from these regulatory documents. We utilized documents from this knowledge base and, combined with prompt engineering, leveraged GPT-4 to generate fine-tuning datasets. This dataset comprises 1488 entries, with 60% (893/1488) allocated to the training set, 30% (446/1488) to the test set, and 10% (149/1488) to the validation set (Table 2).

**Table 2:** Knowledge base and fine-tuning the embedding model dataset.

Dataset	Data source	Data volume
Regulatory Knowledge Base	Regulatory documents issued by the China National Medical Products Administration	29 documents
Embedding Model Fine-Tuning Dataset	Generated using regulatory documents issued by the China National Medical Products Administration	1488

To mitigate the “student-teacher” loop bias introduced by large-model rewriting, we collaborated with the Henan Drug Administration to implement a manual quality control process for all data generated by large models. First, we established a 3-tier annotation standard (perfect equivalence/minor drift/significant drift) and trained annotators (*κ*≥0.78). Next, data were grouped into batches of 100 items for double-blind annotation by pairs of annotators, with a third party serving as the arbitrator. Only when the “significant drift” rate was controlled at ≤5% were the data released. Finally, senior experts centrally reviewed and refined the “significant drift” data.

### Experimental Environment

This work selected Qwen2_7B as the source model and employed a dual-adapter joint fine-tuning approach for training, with specific configurations detailed in Tables 3-5.

**Table 3:** Environmental configuration.

Category	Configuration information
Central processing unit	Xeon(R) Platinum 8352V
Graphics processing unit	RTX 4090 × 2
Graphics memory	24 GB
Distributed training strategy	Data parallelism
Memory	90 GB
Compute Unified Device Architecture	12.2

**Table 4:** Hyperparameter configuration.

Hyperparameter	Value
Train epochs	3
Learning rate	1.00 × 10^–4^
Batch size	2
Lora_rank	32
Logging_steps	3
Gradient accumulation	4

**Table 5:** Retrieval-augmented generation parameter configuration.

Parameters	Value
Chunk size	512
Overlap	80
Top-*k*	5
Cosine	0.72
Nlist	128

### Base Model Testing

This study selected BGE-Large-ZH-V1.5 as the embedding source model. Here, BGE-Large-ZH-V1.5 was compared with M3E-Large, Text2Vec-Large, and Luotuo on the Massive Text Embedding Benchmark (MTEB) and the Chinese Massive Text Embedding Benchmark (C-MTEB). The embedding dimension was unified to 1024, and the evaluation metrics selected were retrieval, semantic textual similarity, pairwise classification, and classification.

Table 6 compares BGE-Large-ZH-V1.5 with mainstream models, demonstrating that it achieves leading performance on both MTEB and C-MTEB. Its high recall score of 70.46 indicates that it is particularly well-suited as a foundation for RAG systems, demonstrating a superior ability to accurately match specialized terminology within knowledge bases compared with other models.

For large models, this paper compares base-model performance by evaluating Qwen2_7B, Llama3_8B, and GLM4_9B across 8 metrics. The results are shown in Table 7.

**Table 6:** Embedding base model test results. This table compares the performance of the BGE-Large-ZH-V1.5 embedding model selected for this work against 3 mainstream Chinese embedding models across multiple dimensions on the MTEBa and C-MTEBb standard evaluation datasets.

Model	Embedded dimension	Retrospective retrieval	Semantic textual similarity	Pairwise classification	Classification
BGE-Large-ZH-V1.5	1024	70.46	56.25	81.6	69.13
M3e-Large	1024	54.75	50.42	64.3	68.2
Text2Vec-Large	1024	41.94	44.97	70.86	60.66
luotuo	1024	44.4	42.78	66.62	61

^a^MTEB: Massive Text Embedding Benchmark.

^b^C-MTEB: Chinese Massive Text Embedding Benchmark.

**Table 7:** Base model test results. This table presents the comparison results of general-purpose models, specialized medical models (HuatuoGPT-7B and DISC-MedLLM), and AE2IN on benchmark metric tests. This evaluation verifies AE2IN’s ability to adapt to specialized domains while preserving foundational general capabilities and assesses its mitigation of catastrophic forgetting.

Indicator/model	AlignBench	MT-Bench	MMLU^a^	GSM8K	MATH	HumanEval	C-Eval	PediaBench
Qwen2_7B	7.21	8.41	70.5	82.3	49.6	79.9	77.2	79.7
Llama3_8B	6.20	8.05	68.4	79.6	30.0	62.2	45.9	59.4
GLM4_9B	7.01	8.35	72.4	79.6	50.6	71.8	75.6	71.5
HuatuoGPT-7B	6.09	8.01	65.1	77.5	28.3	59.3	80.7	88.7
DISC-MedLLM	7.30	8.22	67.3	76.1	45.4	62.1	82.4	90.6
AE2IN	7.32	8.44	67.8	79.2	46.3	64.8	75.1	80.1
Only fine-tuning for specific domains	7.08	8.21	62.5	75.3	39.8	59.4	71.8	73.5

^a^MMLU: Massive Multitask Language Understanding.

Table 7 shows that the Qwen2 model has stronger overall performance than the other 2 models. Qwen2 performs well on the Massive Multitask Language Understanding (MMLU) medical subset and C-Eval medical questions, indicating that the model is stable in diagnostic reasoning and clinical terminology comprehension, but exhibits slight errors in complex calculation questions. Meanwhile, the fine-tuned AE2IN model shows score decreases ranging from 2.1 to 2.7 on the MMLU medical subset and C-Eval medical tasks. Because of the incorporation of PediaBench data during fine-tuning, its scores slightly surpass those of the base model by 0.4 points. By contrast, models fine-tuned solely on domain-specific datasets experienced score declines ranging from 5.4 to 8.0 on the MMLU medical subset and C-Eval medical tasks, along with a 6.2-point drop on PediaBench. These comparative results validate that AE2IN effectively mitigates catastrophic forgetting.

### Comparative Experiment

We selected 5 models for comparative testing with the fine-tuned model. The results are shown in Table 8.

**Table 8:** Comparative evaluation of the fine-tuned model AE2IN against general models and specialized medical models (HuatuoGPT, DISC-MedLLM) in the infant incubator adverse event domain. This table compares the overall performance of the base large model, the base model combined with the same RAGa framework model, mainstream medical-specific large models, and the AE2IN model proposed in this study on the infant incubator adverse event analysis task. All experiments were replicated 3 times independently. Results are presented as mean (SD), supplemented with 2-tailed (unpaired and independent) samples t test *P* values and statistical significance annotations for each model versus AE2IN.

Model	BLEU^b^-4	*P* value	ROUGE-L^c^	*P* value	Accuracy	*P* value	Element recall rate	*P* value	Average IDIAE^d^	*P* value
Qwen2_7B	0.137 (0.031)	<.001	0.208 (0.025)	<.001	0.785 (0.028)	<.001	0.691 (0.023)	<.001	0.61 (0.018)	<.001
Qwen2_7B+RAG	0.162 (0.025)	.003	0.315 (0.022)	.001	0.800 (0.024)	.002	0.715 (0.019)	.002	0.75 (0.015)	.002
Llama3_8B	0.108 (0.026)	<.001	0.237 (0.033)	<.001	0.722 (0.030)	<.001	0.651 (0.028)	<.001	0.49 (0.025)	<.001
Llama3_8B+RAG	0.131 (0.022)	.003	0.302 (0.028)	.002	0.756 (0.026)	.003	0.688 (0.024)	.002	0.62 (0.019)	.003
GLM4_9B	0.105 (0.025)	<.001	0.282 (0.033)	<.001	0.763 (0.031)	<.001	0.636 (0.025)	<.001	0.55 (0.020)	<.001
GLM4_9B+RAG	0.158 (0.021)	.002	0.356 (0.025)	.001	0.801 (0.023)	.002	0.702 (0.021)	.001	0.71 (0.017)	.002
HuatuoGPT-7B	0.155 (0.021)	.03	0.381 (0.022)	.03	0.803 (0.025)	.03	0.722 (0.031)	.03	0.80 (0.022)	.03
DISC-MedLLM	0.174 (0.020)	.02	0.411 (0.024)	.02	0.816 (0.022)	.02	0.740 (0.020)	.02	0.82 (0.019)	.02
AE2IN	0.215 (0.018)	N/A^e^	0.484 (0.021)	N/A	0.898 (0.022)	N/A	0.815 (0.016)	N/A	0.91 (0.013)	N/A

^a^RAG: retrieval-augmented generation.

^b^BLEU: Bilingual Evaluation Understudy.

^c^ROUGE-L: Recall-Oriented Understudy for Gisting Evaluation - Longest Common Subsequence.

^d^IDIAE: Information Density Index of Adverse Events.

^e^N/A: not applicable.

Through systematic quantitative evaluation, the AE2IN model demonstrates notable advantages across all key performance indicators in the domain of infant incubator adverse events. In terms of text generation quality, AE2IN attained a BLEU-4 score of 0.215, reflecting a 56.9% improvement relative to the baseline model and a 32.7% improvement over the baseline equipped with the same RAG framework. Its Recall-Oriented Understudy for Gisting Evaluation - Longest Common Subsequence (ROUGE-L) score reached 0.484, corresponding to a 53.7% increase. For core element analysis of infant incubator adverse events, the element recall rate reached 0.815, representing a 17.9% improvement over the baseline model and a 14.0% improvement over the baseline integrated with the same RAG framework. In the experiments, the dual-adapter architecture exhibited strong causal reasoning capacity when processing complex alarm system failure events.

To conduct a more comprehensive analysis of the AE2IN model, we also selected cases in which accuracy, element recall, and IDIAE were all below 0.5. We found that misjudgment of core elements directly affected completeness conclusions and notably impacted both accuracy and IDIAE values. In failed cases, we observed that for rare fault descriptions—such as the onomatopoeic “pushing clack-clack sound”—low frequency and susceptibility to semantic integrity disruption caused the inferred Query-Key similarity to be drowned out by the attention distribution of primary terms after softmax. This prevented the activation of corresponding neurons during value vector weighting. Furthermore, the scaling coefficient update for (IA)^3^ relies on batch statistics, leading to inconsistent weighting across cases. When encountering such descriptions, the update process may become stuck at a saddle point, failing to amplify this feature.

**Figure 13:**
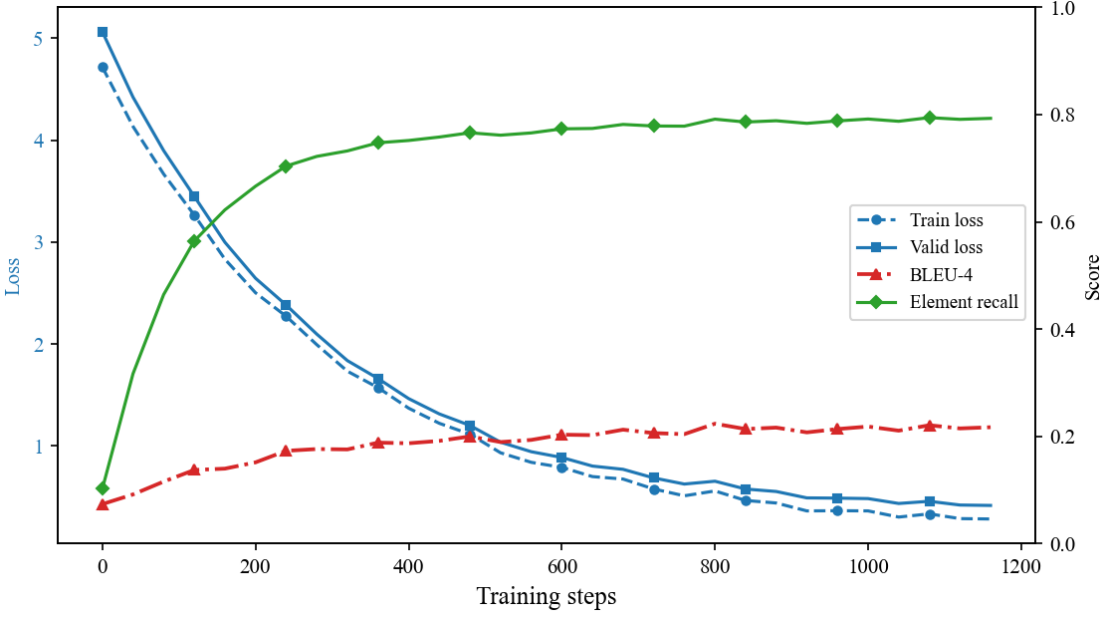
Convergence of dual-adapter fine-tuning (AE2IN). The figure shows the joint evolution of training loss, validation loss, Bilingual Evaluation Understudy-4 score, and ERR metric over training steps. Core hyperparameters were fixed (low-rank adaptation rank=32, learning rate=1 × 10–4, batch size=2). The x-axis represents training steps; the left y-axis shows loss values, and the right y-axis shows text generation and key element extraction scores.

We continuously record training and validation loss, along with key evaluation metrics, to determine in real time whether the model is in a state of normal convergence, overfitting, or underfitting. As shown in Figure 13, validation loss and training loss decrease smoothly and synchronously over the first 600 steps, maintaining a small difference of less than 0.15, indicating that the learning rate of 1 × 10^–4^ effectively balances model capacity and regularization. BLEU-4 increases monotonically from 0.07 to 0.22, only 0.005 away from the final test set score of 0.215, confirming that the validation and test sets are identically distributed and that the model has fully converged. Feature recall also increases from 0.60 to 0.815 over the first 600 steps, rising in parallel with BLEU-4 without intersecting, indicating that the model improves fluency without sacrificing its ability to extract key information. All 3 metrics plateau simultaneously, demonstrating stable convergence without the need for early stopping or secondary parameter tuning. This also indicates that the final test score is not a random peak but the result of stable convergence.

**Figure 14:**
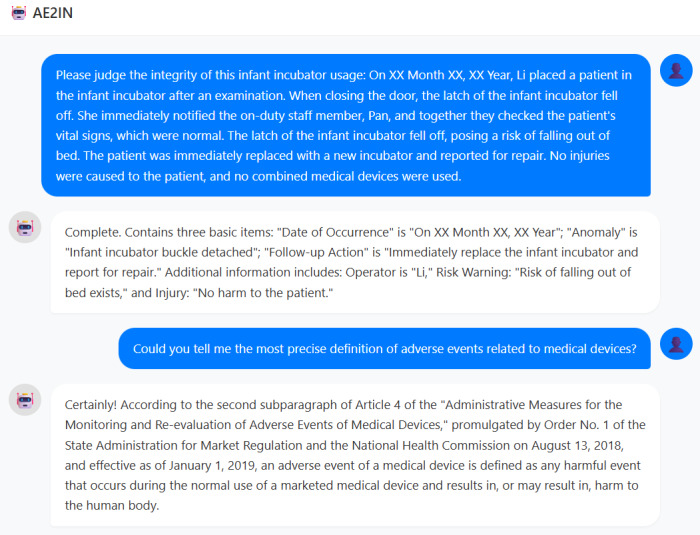
AE2IN response examples. The figure presents sample outputs from AE2IN, including assessments of adverse event report completeness for infant incubators and responses to medical device adverse event definitions.

As shown in Figure 14, when determining the completeness of adverse events related to infant incubators, AE2IN directly extracts core elements—incident date, nature of the abnormality, and subsequent corrective actions—while supplementing them with details such as operator information, risk warnings, and injury status. This approach concisely highlights essential information.

However, compared with other models, AE2IN’s responses tend to be more concise. To investigate whether this brevity affects safety, a supplementary risk recall assessment experiment was conducted to quantify the quality of model outputs from a safety perspective. First, a core safety item list was established through expert consultation and alignment with the “Measures for the Administration of Medical Device Adverse Event Monitoring and Re-evaluation” and the national standard “GB 9706.219-2021.” This list encompasses adverse event phenomenon identification, safety risk identification, immediate patient safety measures, equipment disposal measures, adverse event reporting measures, and risk cause localization. Next, 100 high-quality data entries, each containing core safety items, were extracted and annotated, and the process was repeated 5 times. The results of the risk recall experiment are shown in Table 9.

**Table 9:** Risk recall rate comparison. This table displays the risk recall rates and the average number of core safety items identified by AE2IN across various models.

Model	Risk recall, mean (SD)	Average number of core safety items identified
AE2IN	0.92 (0.012)	5.52
Qwen2_7B	0.77 (0.024)	4.62
Llama3_8B	0.75 (0.022)	4.50
GLM4_9B	0.80 (0.019)	4.80
HuatuoGPT-7B	0.82 (0.016)	4.92
DISC-MedLLM	0.84 (0.017)	5.04

AE2IN demonstrates the highest risk recall rate, achieving an improvement of approximately 15% over other models. Although some data may be underreported due to rare fault descriptions, its overall risk recall rate confirms that AE2IN effectively maximizes risk extraction. By outputting structured information that highlights core details, it helps ensure safety.

We used the AE2IN model with the same RAG framework and different embedding models for regulatory question answering. The experiments used a regulatory clause validation set to compare the performance of the fine-tuned embedding models. The comparative results are shown in Table 10.

**Table 10:** Comparative evaluation of regulatory question-answering performance between the AE2IN model with different embedding models and general-purpose models.

Configuration/indicators	AE2IN						General model		
	FINBGE^a^ (ours)	BGE-Large-ZH-V1.5	M3e-Large	Text2Vec-Large	Luotuo	Qwen2_7B		Llama3_8B	GLM4_9B
Accuracy, mean (SD)	0.938 (0.011)	0.863 (0.015)	0.804 (0.015)	0.780 (0.016)	0.761 (0.017)	0.715 (0.038)		0.652 (0.040)	0.727 0.042)
Recall, mean (SD)	0.894 (0.013)	0.807 (0.016)	0.763 (0.017)	0.759 (0.017)	0.685 (0.019)	0.652 (0.042)		0.594 (0.044)	0.671 (0.050)
*F*_1_-score, mean (SD)	0.915 (0.013)	0.834 (0.016)	0.783 (0.016)	0.769 (0.017)	0.721 (0.018)	0.682 (0.041)		0.622 (0.038)	0.698 (0.04)

^a^FINBGE is a BGE model fine-tuned using supervised contrastive learning.

The experimental results in Table 11 show that the AE2IN model, when equipped with different embedding models, exhibits a clear performance gradient in the regulatory question-answering task. The FINBGE model leads by a notable margin, achieving the highest levels across the 3 core metrics—accuracy, recall, and *F*_1_-score—compared with the source model BGE-Large-ZH-V1.5. This result validates the effectiveness of the supervised contrastive semantic optimization fine-tuning strategy, which successfully narrows the vector distance between the question and the correct answer. Furthermore, comparison with common models reveals that Llama3_8B achieves the lowest scores, likely because the test dataset consists entirely of Chinese regulatory question-answer pairs. The best-performing general model, GLM4_9B, achieves scores of 0.727, 0.671, and 0.698 in accuracy, recall, and *F*_1_-score, respectively, but still lags behind AE2IN. This demonstrates the necessity of domain adaptation and the RAG architecture, which can mitigate model hallucinations, address knowledge timeliness, and improve accuracy.

**Table 11:** AE2IN model performance ablation study.

Indicators/configuration	BLEU^a^-4, mean (SD)	ROUGE-L^b^, mean (SD)	Accuracy, mean (SD)	Element recall rate, mean (SD)	IDIAE^c^, mean (SD)
AE2IN	0.215 (0.018)	0.484 (0.021)	0.898 (0.022)	0.815 (0.016)	0.91 (0.01)
Only LoRA^d^	0.187 (0.015)	0.412 (0.019)	0.824 (0.020)	0.763 (0.015)	0.83 (0.01)
Only (IA)^3e^	0.176 (0.015)	0.398 (0.017)	0.795 (0.018)	0.741 (0.014)	0.79 (0.01)
Full fine-tuning	0.168 (0.022)	0.388 (0.024)	0.802 (0.028)	0.735 (0.021)	0.78 (0.02)
None	0.137 (0.031)	0.208 (0.025)	0.785 (0.028)	0.691 (0.023)	0.61 (0.02)

^a^BLEU: Bilingual Evaluation Understudy.

^b^ROUGE-L: Recall-Oriented Understudy for Gisting Evaluation - Longest Common Subsequence.

^c^IDIAE: Information Density Index of Adverse Events.

^d^LoRA: low-rank adaptation.

^e^(IA)^3^: Infused Adapter by Inhibiting and Amplifying Inner Activations.

Similarly, to conduct a more comprehensive analysis of the system model, we selected cases in which regulatory question-answering accuracy was below 0.5 for further study. We found that hallucinations still occurred—for instance, when the model should have referenced article 23 of the “Administrative Measures for the Monitoring and Re-evaluation of Adverse Events of Medical Devices,” it instead cited article 24. This occurs because clauses with high similarity but different article numbers exhibit only minor differences in cosine similarity, leading to the retrieval of incorrect clauses and subsequently triggering hallucinations.

### Ablation Experiments

To validate the contributions of each component in the dual-adapter joint fine-tuning architecture, this study designed a systematic ablation experiment, maintaining the same testing environment and hyperparameter settings as previously described. The experiment employed a controlled variable approach, sequentially removing key components and observing changes in model performance.

Table 11 shows that the complete model improves feature recall by an average of 9.25% compared with the single-adapter solution, validating the complementarity of low-rank updates and feature scaling in equation (1).

When the LoRA component is removed, BLEU-4 and ROUGE-L decrease notably. This is mainly because LoRA optimizes the long-range dependency modeling capability of the Transformer layer through low-rank decomposition. Its absence makes it difficult for the model to effectively capture the logic in infant incubator failure events. At the same time, the feature recall rate decreases by 10.0%, further validating LoRA’s key role in structured information extraction—its trainable parameters are concentrated in the Δ*W* matrix of the attention layer, enabling more accurate localization of core elements in fault descriptions.

When the (IA)^3^ component is removed, the IDIAE index decreases notably, due to the weakening of the dynamic feature-scaling function achieved by (IA)^3^ through the gating mechanism. Specifically, the key-value intervention vector in (IA)^3^ suppresses the activation of irrelevant features while enhancing the response strength of neurons associated with medical terms.

Following full fine-tuning, metrics exhibited varying degrees of decline compared with AE2IN or single-method fine-tuning. This phenomenon stems from the inevitable overfitting of the 7-billion-parameter model on finite, small samples, validating the necessity of structured parameter fine-tuning for efficiency in small-sample scenarios.

**Figure 15:**
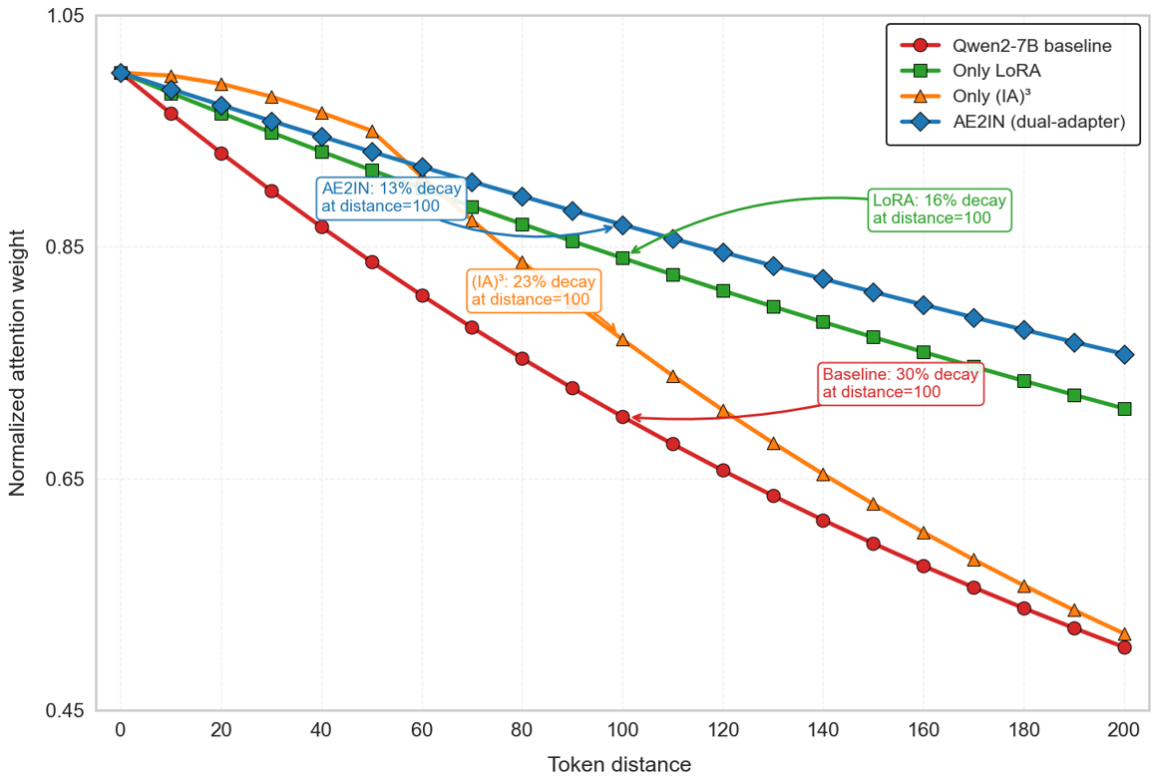
Dual-adapter semantic modeling capability. The figure demonstrates the model’s ability to capture semantics using a long-text test dataset of infant incubator adverse events. It shows the decay of attention weights with increasing token distance: the x-axis represents token distance, and the y-axis shows normalized attention weights, comparing long- and short-range semantic capture across four architectures. (IA)3: Infused Adapter by Inhibiting and Amplifying Inner Activations; LoRA: low-rank adaptation.

Observation of Table 12 data reveals that, in long-text scenarios, the recall rate of the LoRA configuration alone exhibits a lower decay rate than (IA)^3^, demonstrating that LoRA’s low-rank updates effectively capture long-range semantic associations across clauses. Figure 15 further shows that the attention weights in the LoRA branch decay by 16% for long-distance word pairs, representing a 14-percentage-point reduction compared with the baseline. This indicates that LoRA effectively enhances the interaction strength of distant semantic units. (IA)^3^ can boost weights over short distances, but long-range dependencies are easily lost, leading to reduced recall rates. This also explains why, under dual-adapter collaboration, the final performance in the infant incubator adverse-event domain improved by approximately 14%, further demonstrating that the 2 adapters can complement each other across different distance scales.

**Table 12:** AE2IN element recall rate ablation experiment results at different input lengths. This table compares the element recall rate of different model architectures across short and long text adverse event reports for infant incubators.

Configuration	Short text (≤64 tokens), mean (SD)	Long text (>128 tokens), mean (SD)	Attenuation level (%)
AE2IN	0.847 (0.012)	0.810 (0.019)	4.4 (1.1)
Only LoRA^a^	0.834 (0.015)	0.766 (0.024)	8.2 (1.8)
Only (IA)^3b^	0.829 (0.018)	0.739 (0.029)	10.9 (2.2)
Full fine-tuning	0.781 (0.021)	0.704 (0.032)	9.9 (2.6)
None	0.733 (0.028)	0.661 (0.035)	9.7 (3.1)

^a^LoRA: low-rank adaptation.

^b^(IA)^3^: Infused Adapter by Inhibiting and Amplifying Inner Activations.

## Discussion

### Principal Findings

This study proposes AE2IN, a large-model framework that integrates RAG with dual-adapter joint fine-tuning for the intelligent analysis of adverse events in infant incubators. Experimental results demonstrate that this framework significantly outperforms baseline models across key metrics, including text generation quality, element recall rate, and regulatory question-and-answer accuracy. Under the same experimental conditions, the element recall rate reaches 0.815, the accuracy of infant incubator adverse event analysis is 0.898, and the accuracy in regulatory clause question-answering tasks attains 0.938. The dual-adapter architecture, that is, LoRA + (IA)^3^, effectively infuses domain knowledge while preserving model generalization, thereby mitigating catastrophic forgetting. The RAG module, combined with the FINBGE embedding model optimized through supervised contrastive semantic learning, significantly enhances knowledge retrieval accuracy and reduces hallucination.

### Comparison With Prior Work

Compared with traditional deep learning models, AE2IN demonstrates superior contextual modeling and causal reasoning capabilities, making it particularly well-suited for complex tasks involving multiple intertwined factors and specialized terminology, such as adverse events in infant incubators. Compared with single fine-tuning approaches, the dual-adapter architecture proposed in this paper achieves a better balance between parameter efficiency and task adaptability. Compared with existing RAG medical applications, AE2IN further introduces a domain-specific embedding model fine-tuning mechanism, enhancing the matching accuracy of Chinese regulatory clauses. When benchmarked against general models such as Qwen and Llama, AE2IN demonstrates higher professional consistency and stronger element-extraction capabilities in specialized tasks, particularly excelling in structured output and terminology standardization.

### Limitations

First, the dataset size is limited. The infant incubator adverse-event dataset used in this study comprises 2530 entries. Despite undergoing joint cleaning and enhancement by human experts and GPT-4, the dataset remains insufficient for rare fault types or complex scenarios. To mitigate this issue, we introduced PediaBench general pediatric data for mixed training, employing a 3:1 domain-to-general data sampling ratio to enhance generalization. Future efforts could expand multicenter, multiyear data through collaborations with additional medical institutions to further improve model robustness. Second, the regulatory knowledge base updates lag. The regulatory knowledge base used in this study cannot guarantee real-time coverage of the latest policy revisions. We partially mitigated this knowledge lag by improving the embedding model’s semantic understanding of clauses through supervised contrastive fine-tuning. Future work should consider establishing a dynamic regulatory update mechanism, such as periodically crawling the National Medical Products Administration website or integrating official application programming interfaces to enable automatic knowledge base synchronization. Third, prospective clinical validation was not conducted. This retrospective study did not verify the model’s usability and safety within actual clinical workflows. We collaborated with the Henan Drug Administration to implement manual quality control, maintaining a “significant drift” rate ≤5%. However, there remains a lack of assessment regarding clinicians’ adoption of model recommendations and the impact of false positives. Future prospective studies should be conducted, inviting hospital equipment departments or adverse-event monitoring personnel to participate in pilot programs and collecting user feedback for iterative optimization.

### Future Directions

Current research is limited to adverse events involving infant incubators. Future studies may further explore data-level approaches, such as constructing larger-scale, multicenter, multilingual adverse-event databases covering more high-risk medical device types to enhance model generalizability. Then, at the fine-tuning method level, future work could draw inspiration from distributed entropy regularization [37] to further optimize the learning objective of scaling factors, thereby improving sensitivity to uncommon failure patterns in the training data. At the model level, multimodal fusion could be explored by incorporating visual encoders to process device alarm screenshots or failure photographs, enabling more comprehensive fault identification. Finally, at the system level, an integrated regulatory support system should be developed. This system would integrate the AE2IN model, regulatory retrieval, report generation, and manual review workflows to support multiparty collaboration among hospital equipment departments, regulatory agencies, and manufacturers, thereby advancing the implementation of intelligent regulation.

### Conclusions

The safety management of medical devices encompasses all stages of the product life cycle, including research and development, design, manufacturing, clinical application, maintenance, and product recalls, all of which require strict quality control measures. By establishing standardized protocols, refining risk-prevention mechanisms, conducting continuous monitoring, and enhancing professional training, the failure rate of medical devices can be significantly reduced, thereby effectively safeguarding the safety interests of both medical professionals and patients. To advance the management of adverse events related to medical devices and comprehensively support professionals in this field—such as surveillance personnel—this study proposes a large-model solution that integrates RAG technology with dual-adapter fine-tuning. This approach provides an efficient and reliable technical pathway for analyzing adverse events involving infant incubators.

## Data Availability

These data contain confidential information. To access them, authorization must be requested from the corresponding author (PZ).

## Appendix

Multimedia Appendix 1 Example of raw data for adverse events in infant incubators.

## Abbreviations

(IA)3: Infused Adapter by Inhibiting and Amplifying Inner Activations
AI: artificial intelligence
BERT: Bidirectional Encoder Representations from Transformers
BLEU: Bilingual Evaluation Understudy
C-MTEB: Chinese Massive Text Embedding Benchmark
FDA: Food and Drug Administration
IDIAE: Information Density Index of Adverse Events
LLM: large language model
LoRA: low-rank adaptation
MAUDE: Manufacturer and User Facility Device Experience
MMLU: Massive Multitask Language Understanding
MTEB: Massive Text Embedding Benchmark
Q/V: query/value
RAG: retrieval-augmented generation
ROUGE-L: Recall-Oriented Understudy for Gisting Evaluation - Longest Common Subsequence
